# Transcatheter Embolotherapy of Maternal Pulmonary Arteriovenous Malformation During Twin Pregnancy With Hereditary Hemorrhagic Telangiectasia: A Case Report

**DOI:** 10.7759/cureus.106913

**Published:** 2026-04-12

**Authors:** Akiko Takashima, Tomomi Komiyama, Megumi Manrai, Shusuke Kasuya, Hitoshi Terada

**Affiliations:** 1 Obsterics and Gynecology, Toho University Sakura Medical Center, Sakura, JPN; 2 Obstetrics and Gynecology, Toho University Sakura Medical Center, Sakura, JPN; 3 Oncology, Toho University Sakura Medical Center, Sakura, JPN; 4 Radiology, Toho University Sakura Medical Center, Sakura, JPN

**Keywords:** hereditary hemorrhagic telangiectasia (hht), high-risk pregnancy, pulmonary arteriovenous malformations, spontaneous rupture, transcatheter embolotherapy

## Abstract

Hereditary hemorrhagic telangiectasia (HHT) is a multiple-organ disease. Herein, we report a case of rupture of an undiagnosed pulmonary arteriovenous malformation (PAVM) during pregnancy in which good outcomes for both mother and children were achieved with transcatheter embolization.

The patient was a pregnant 32-year-old Pashtun woman, G4P3, with a history of cesarean section. She had undergone pregnancy checkups at our hospital for a dichorionic diamniotic twin pregnancy. In gestational week 20, the patient experienced loss of consciousness at home and was transported by ambulance to our hospital. Her Glasgow Coma Scale score was 9 (E2V2M5) and her blood pressure was 57/27 mmHg. Blood test results showed severe anemia; right atelectasis and mediastinal shift were shown on chest radiography. Contrast-enhanced chest computed tomography revealed right hemothorax and a single pulmonary arteriovenous malformation measuring 35 mm in the middle lobe of the right lung. The patient was diagnosed with hemothorax due to rupture of the right PAVM. Emergency transcatheter embolization was performed, disappearance of the arteriovenous malformation was confirmed, and hemostasis was achieved. Recurrent epistaxis and telangiectasias on the tongue, buccal mucosa, and nasal cavity were noted, and the patient was diagnosed with HHT. An abdominal cesarean section was performed due to the low-lying placenta and right hydronephrosis. Two male neonates were delivered: one weighed 1988 g and the other 2166 g. Both mother and infants progressed well.

This case illustrates how unrecognized HHT can lead to catastrophic PAVM rupture during pregnancy and emphasizes the lifesaving value of early diagnosis and prompt endovascular intervention. In addition, it highlights the critical importance of pre‑pregnancy screening for HHT to prevent life‑threatening complications.

## Introduction

Hereditary hemorrhagic telangiectasia (HHT) is an autosomal dominant bleeding disorder caused by malformed vessels. HHT affects 1 in 5000-8000 individuals and can affect both genders and people of all races [1]. The Curaçao criteria are four clinical diagnostic criteria for HHT: 1) spontaneous and recurrent epistaxis, 2) multiple telangiectasias at characteristic sites (lips, oral cavity, fingers, nose), 3) visceral arteriovenous malformations (pulmonary, hepatic, cerebral, spinal, or gastrointestinal), and 4) family history: diagnosis of HHT in a first-degree relative. The presence of three criteria confirms a definite diagnosis, two criteria indicate possible HHT, and fewer than two criteria make HHT unlikely [2].

 HHT is a mucocutaneous and fibrovascular dysplasia, and the spontaneous rupture of pulmonary arteriovenous malformations (PAVMs), resulting in pulmonary hemorrhage and hemothorax, represents a life-threatening complication. Consequently, PAVMs are regarded as one of the most feared manifestations of HHT. Pregnancy is known to exacerbate vascular lesions such as PAVMs due to increases in blood volume, cardiac output, and venous distensibility [1]. Despite these recognized risks, evidence remains limited regarding the optimal emergency management of PAVM rupture during pregnancy, particularly in multifetal gestations, where hemodynamic stress is even greater. This gap underscores the need for detailed case reports to guide clinical decision‑making. Given these risks, reports of PAVM rupture during pregnancy are especially valuable for elucidating clinical warning signs and informing appropriate emergency management. Here, we describe a case of a woman at 20 weeks of dichorionic diamniotic twin gestation who experienced spontaneous rupture of a right PAVM and underwent successful transcatheter embolization. Although PAVM rupture during pregnancy is exceedingly rare, reports involving twin pregnancies and their interventional management are particularly scarce. To our knowledge, published descriptions of embolization performed in the setting of a twin pregnancy are extremely limited. This case, therefore, provides important insight into the acute management of this high‑risk scenario.

## Case presentation

A 32-year-old Pashtun woman experienced a sudden loss of consciousness at home. The woman, G4P3, had a personal history of three previous abdominal cesarean sections. She was 155 cm tall, weighed 59.7 kg, and had a body mass index of 24.8 kg/m2. Her family history was unknown.

The history of our patient's presenting illness included a checkup in the first trimester of the pregnancy, at which time a dichorionic diamniotic twin pregnancy was identified.

**Figure 1:**
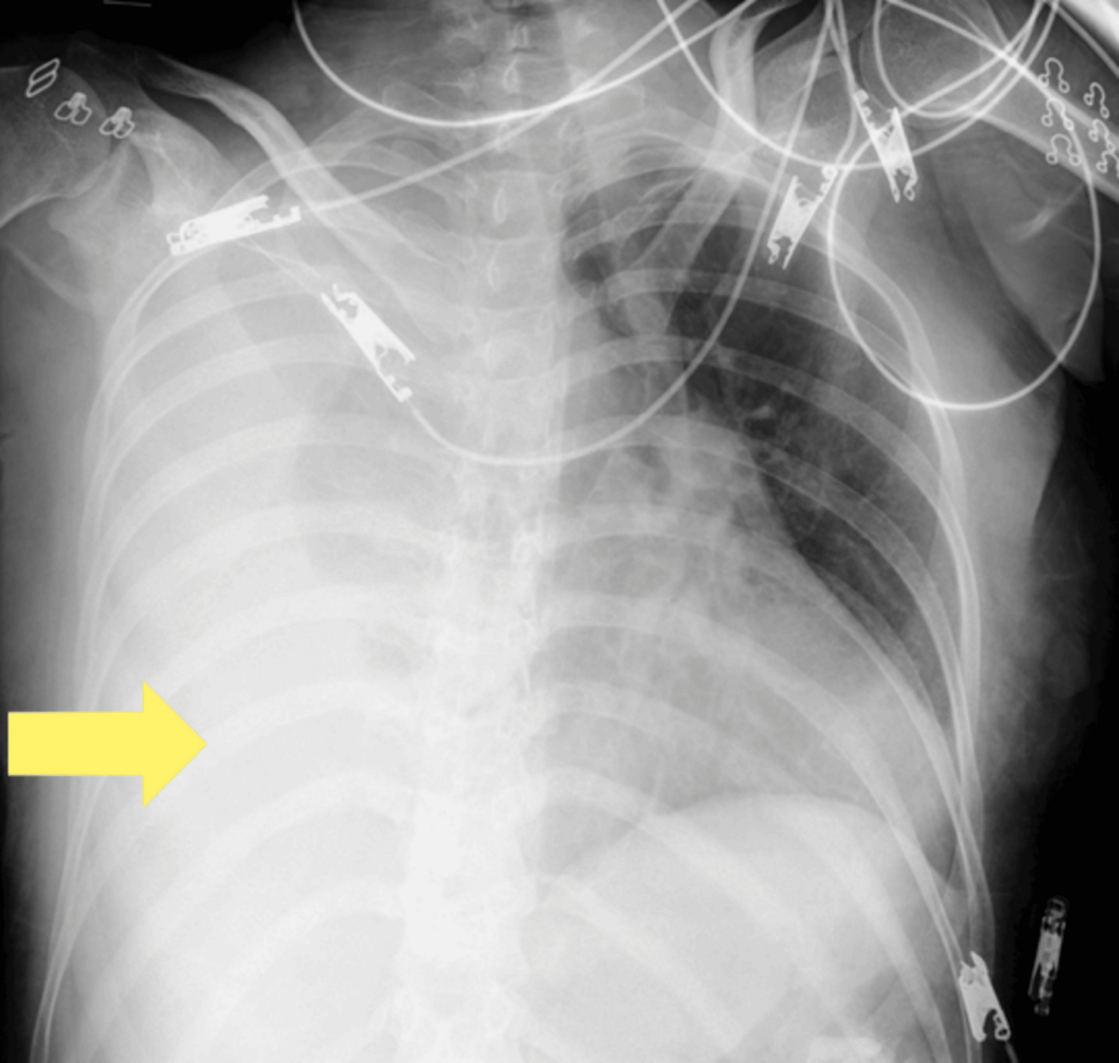
Chest radiograph Chest radiograph showing mediastinal shift and right atelectasis.

**Figure 2:**
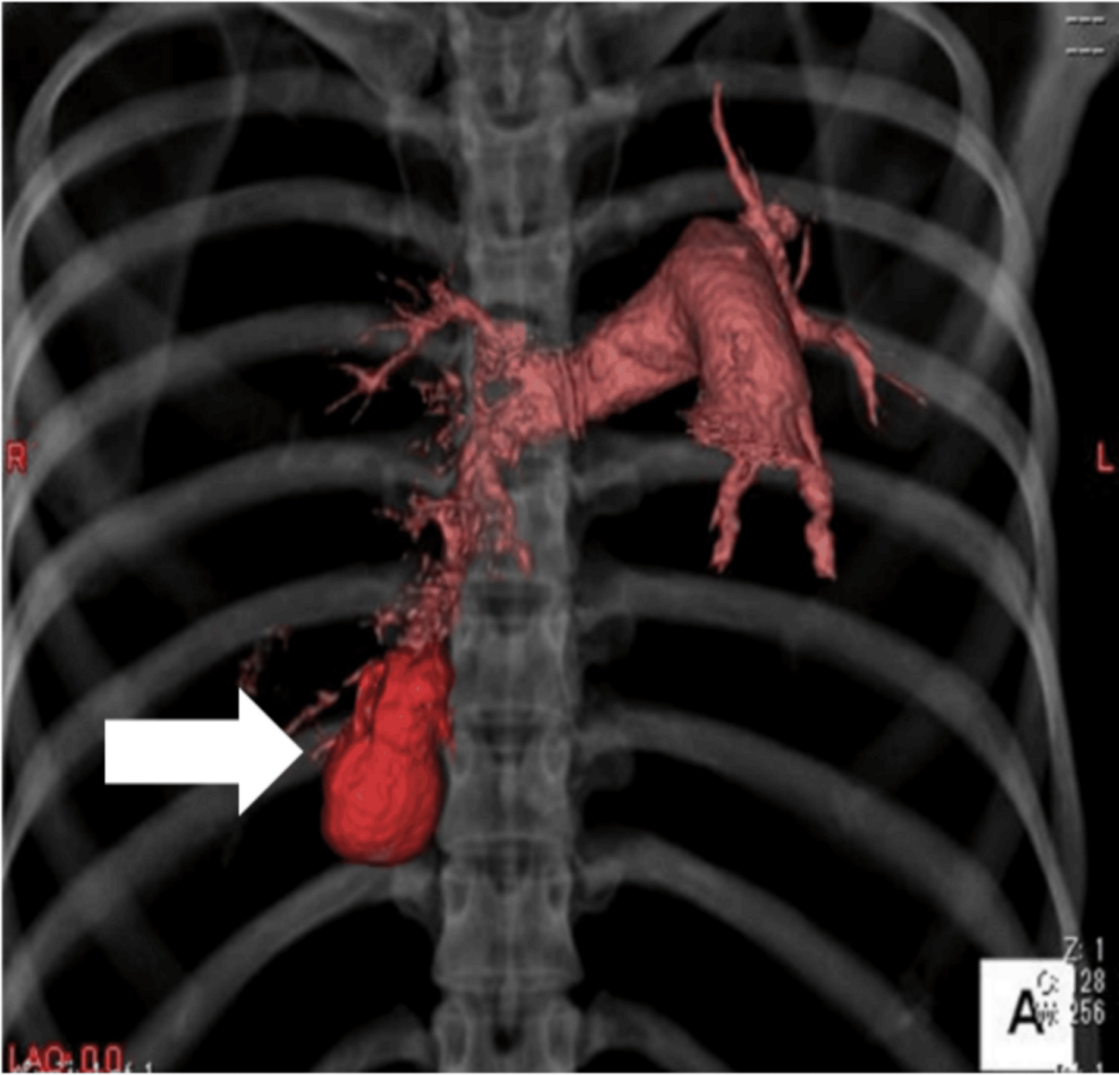
Contrast-enhanced chest computed tomography scan Contrast-enhanced chest computed tomography scan showing the pulmonary arteriovenous malformation (arrow).

**Figure 3:**
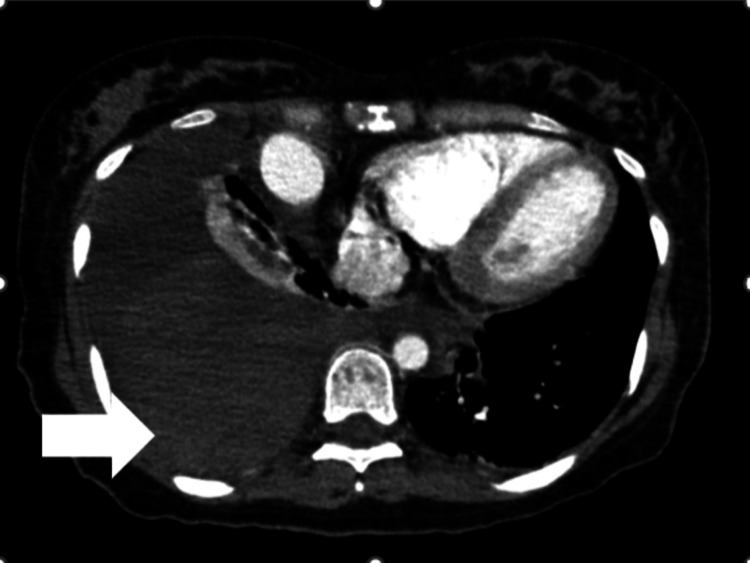
Contrast-enhanced chest computed tomography scan Contrast‑enhanced axial chest CT showing a substantial right‑sided hemothorax. High‑density coagulated blood products are seen layering dependently along the dorsal thoracic cavity (arrow).

At gestational week 20 day four, the patient became unconscious at home and was transported by ambulance. Her Glasgow Coma Scale score was 9 (E2V2M5), blood pressure 57/27 mmHg, pulse 82 beats per minute, body temperature 37.2 ℃, oxygen saturation 97 % on room air, and Sequential Organ Failure Assessment score 5 points. Blood test results revealed a hemoglobin level of 5.9 g/dL (Table 1). The patient was diagnosed with impaired consciousness and hypotension due to severe anemia and was immediately admitted for treatment. In the differential diagnosis of loss of consciousness accompanied by severe anemia, cerebrovascular and hemorrhagic etiologies were considered, and the following diagnostic evaluations were undertaken. No abnormalities were noted on the head computed tomography (CT). Because the fetus was completely outside the irradiation field, the estimated fetal radiation exposure was negligible, and abdominal lead shielding was applied during the examination. Electrocardiography and chest radiography were performed due to the onset of chest pain; the findings of these examinations revealed right atelectasis and mediastinal shift (Figure 1). A contrast-enhanced CT examination was urgently performed, and a rupture of a right PAVM was identified on the scan (Figure 2), leading to a diagnosis of right hemothorax (Figure 3). After a comprehensive differential diagnostic evaluation, the patient's hypotension was ultimately attributed to severe anemia, and four units of packed red blood cells were transfused. A multidisciplinary team comprising obstetricians, pediatricians, respiratory physicians, and interventional radiologists held several discussions to determine the most appropriate management strategy. Given the patient's pregnancy and considering the maintenance of vital signs, minimally invasive transcatheter embolization was performed. Given the patient's pregnancy and stable vital signs, transcatheter embolization was performed under local anesthesia with minimal sedation. Fetal heart rates were assessed immediately before and after the procedure to avoid unnecessary radiation exposure during the intervention.

**Table 1:** Laboratory findings at presentation and pre‑procedural evaluation FDP - fibrin degradation products

Timing	Laboratory test	Result	Reference range	Unit
At presentation (GW 20+4)	Hemoglobin	5.9	11.5-15.0	g/dL
Pre‑embolization (GW 20+5)	Hemoglobin	7.6	11.5-15.0	g/dL
Pre‑embolization (GW 20+5)	FDP	10.2	2.0-8.0	µg/mL
Pre‑embolization (GW 20+5)	Fibrinogen	634	200-400	mg/dL

**Figure 4:**
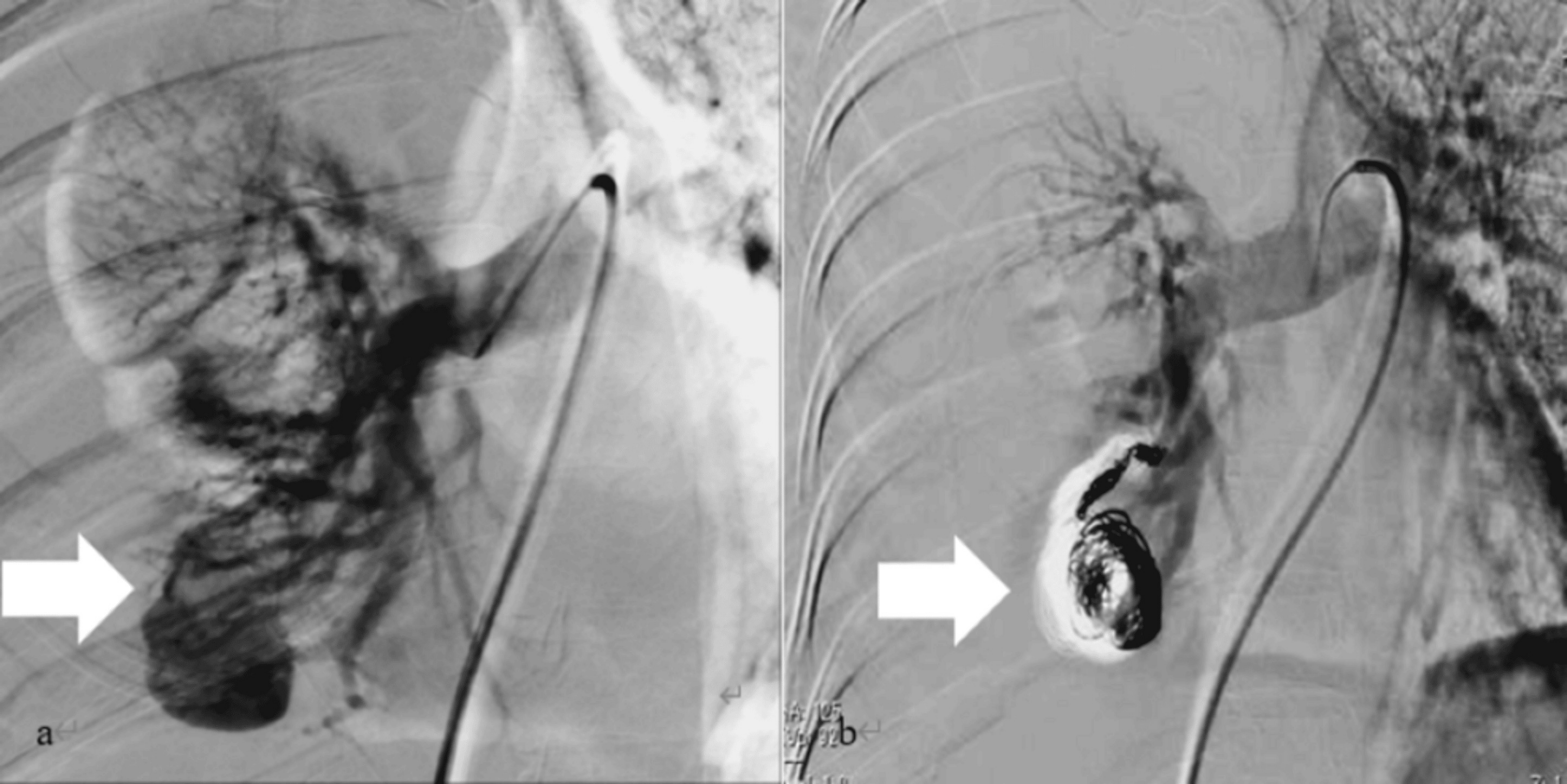
Angiographic findings before and after transcatheter embolization (a) The radiograph shows a pulmonary arteriovenous malformation arising from the right branch of the middle lobar artery. A large aneurysmal sac measuring 25 × 30 mm, with no evidence of intrathoracic leakage of the contrast agent, is visible (arrow). The findings were interpreted as massive hemothorax and tamponade hemostasis due to hematoma. (b) After transcatheter embolization, the early venous return has disappeared, and hemostasis is confirmed (arrow).

At gestational week 20 day five, presurgical blood test findings were a hemoglobin level of 7.6 g/dL, FDP of 10.2 µg/dL, and fibrinogen level of 634 mg/dL; no coagulation abnormalities resulting in critical bleeding were noted (Table 1). Considering the risk of sudden changes, four units of red blood cells and four units of platelet concentrate were transfused during the procedure. The transcatheter embolization procedure was as follows: Using a right femoral vein approach, the right middle lobar artery was selected. Early venous return and a large venous sac measuring 25 × 30 mm were visualized in the imaged area (Figure 4a). No intrathoracic leakage of the contrast agent occurred, and the findings were interpreted as massive hemothorax and tamponade hemostasis due to hematoma. The venous sac was successfully embolized using detachable coils; contrast-enhanced imaging of the right pulmonary artery trunk confirmed the disappearance of the arteriovenous malformation, and the procedure was concluded (Figure 4b). No signs of repeated rupture were observed after the surgery, and the patient was discharged to the intensive care unit three days later (gestational week 21 day one). The postoperative clinical course of the patient is described below.

**Figure 5:**
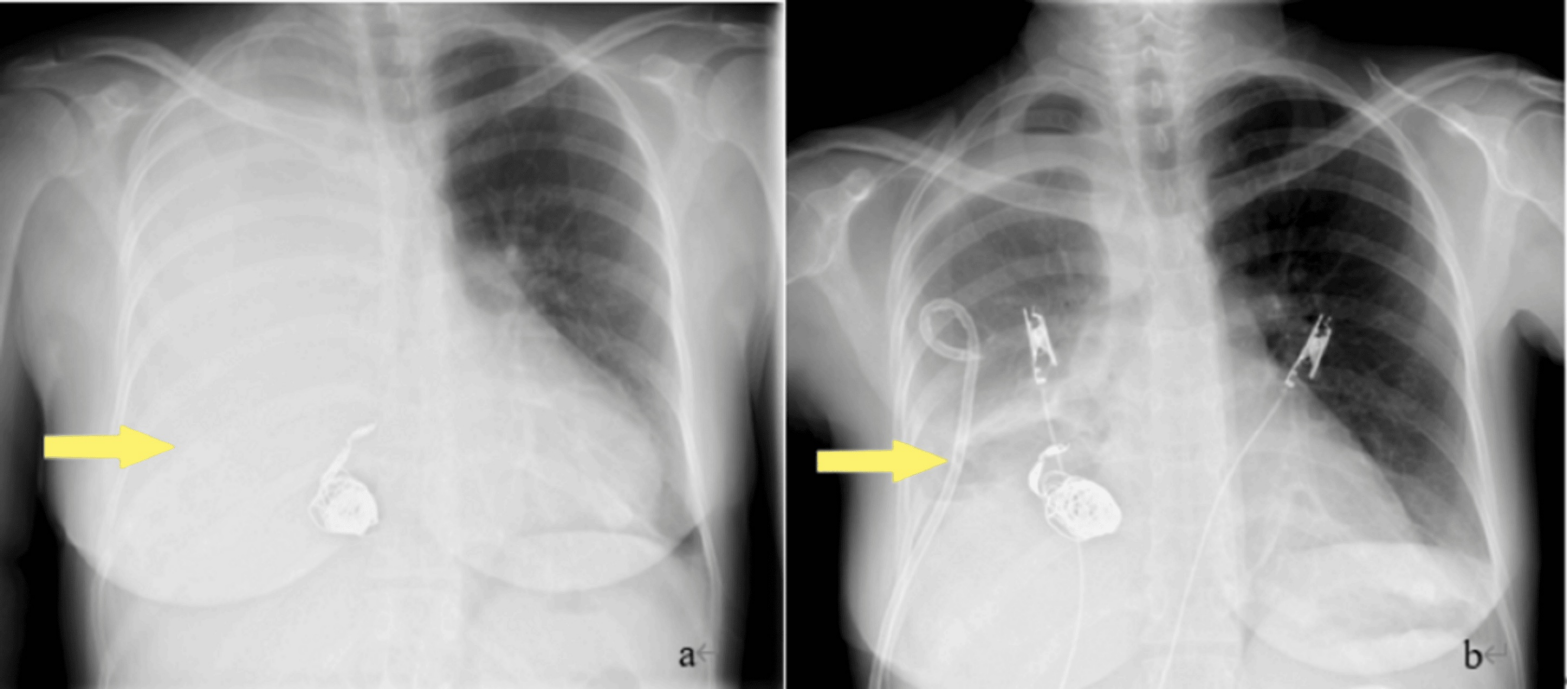
Postoperative chest radiographs (a) Ten days after surgery, mediastinal shift is observed. (b) Three days after inserting a pigtail drainage catheter, improvement is observed.

At gestational week 22 day one, a chest radiograph revealed worsening mediastinal shift (Figure 5a), but chest CT confirmed no repeated rupture. The patient was diagnosed with mediastinal shift due to hemorrhagic pleural effusion, and a 14 French pigtail drainage catheter was inserted from the right side of the chest. At gestational week 23 day one, improvement of the pleural effusion was noted (Figure 5b), and the thoracic drain was removed.

**Figure 6:**
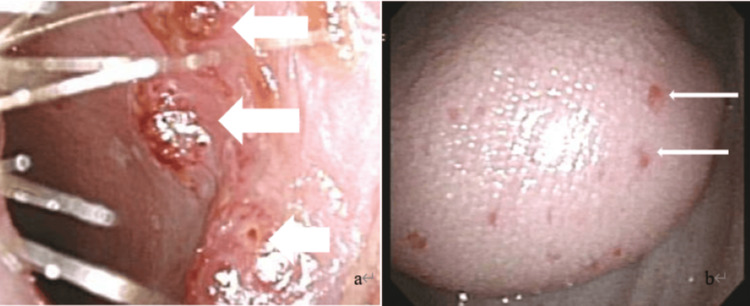
Otolaryngology examination findings (a) Multiple nodular lesions are visible in the nasal mucosa (arrows). (b) Multiple nodular lesions are visible in the tongue mucosa (arrows).

In light of the clinical suspicion of HHT, the patient was referred for further diagnostic evaluation to establish a definitive diagnosis. A detailed medical history was taken, and revealed that spontaneous and recurrent epistaxis and mucocutaneous telangiectasia had also been noted during previous pregnancies. However, no further diagnostic evaluation was performed at that time, and a definitive diagnosis of HHT was not established until the current pregnancy. The patient was examined in the otolaryngology department, and telangiectasias were noted on the tongue and in the buccal mucosa and nasal cavity (Figure 6). An abdominal ultrasound study found no hepatic vascular malformations, but several hyperechoic nodules were shown, suggesting nodular hyperplasia due to hepatic blood flow abnormalities. A head magnetic resonance imaging examination was performed, and the findings confirmed that no preexisting intracranial vascular malformations or paradoxical embolisms were present. Because three of the Curaçao Criteria (spontaneous and recurrent epistaxis, oral cavity and nose telangiectasias, PAVMs) were thus met, the patient was diagnosed with HHT. The patient was discharged home with instructions to measure her oxygen saturation levels at home. She attended weekly outpatient checkup visits thereafter, and good progression was confirmed despite a low-lying placenta.

At gestational week 28 day four, follow-up imaging was performed for postoperative monitoring. Lumbar magnetic resonance imaging demonstrated no evidence of lumbar arteriovenous malformations and indicated that spinal anesthesia could be safely performed. Chest radiography was also performed, and revealed residual but improving atelectasis.

The patient was admitted from gestational week 34 day three to start treatment for post-PAVM procedure management and low-lying placenta. During admission, 400 mL of autologous blood was prepared for potential use at delivery.

At gestational week 35 day four, the patient experienced a sudden onset of intense low back pain and, based on the findings of abdominal ultrasound and CT scans, she was diagnosed with right hydronephrosis resulting from the pregnant uterus. Because the pain was refractory to medical management, an urgent cesarean section was performed on the same day. This was the patient's fourth cesarean delivery. Given her underlying HHT and the cumulative maternal risks associated with repeat cesarean delivery, a tubal ligation was performed concurrently. The first infant delivered had a birth weight of 1988 g (-1.44 SD), Apgar scores of 8 and 9 points at one and five minutes, respectively, and a cord blood gas pH of 7.196. The second infant delivered had a birth weight of 2166 g (-0.88 SD), Apgar scores of 8 and 9 points at one and five minutes, respectively, and a cord blood gas pH of 7.353.

After delivering the placenta, continuous bleeding from the placental separation surface was noted. A balloon catheter was inserted into the uterine cavity, and the procedure was concluded. The duration of surgery was one hour and 22 minutes, with a hemorrhage volume of 1257 mL, including amniotic fluid. The newborns were admitted to the Neonatal Intensive Care Unit because their births were preterm and their birth weights were low. Both infants required short-term respiratory support with nasal continuous positive airway pressure for transient respiratory insufficiency, as well as intravenous glucose supplementation for hypoglycemia. No additional complications occurred during their NICU stay. One day after the surgery, the balloon catheter was removed from the uterine cavity. As the patient's post-surgical course was good, she was discharged five days after surgery.

Neonatal outcomes were favorable, with no evidence of adverse effects attributable to maternal diagnostic procedures or therapeutic interventions. Both infants showed steady improvement, were weaned off respiratory support within a few days, and were discharged home 19 days after birth (corrected gestational age, 38 weeks and two days).

**Figure 7:**
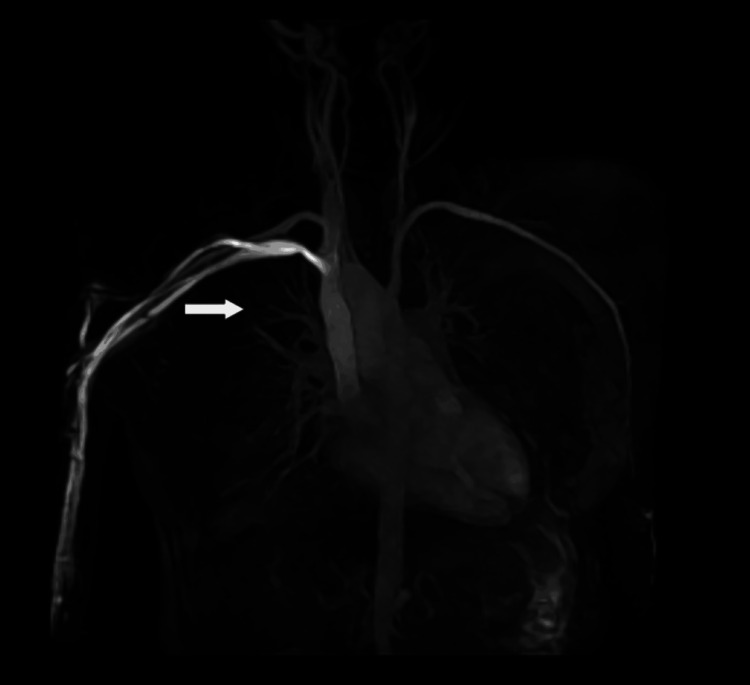
Contrast-enhanced chest magnetic resonance image Contrast-enhanced chest magnetic resonance image showing that the pulmonary arteriovenous malformation has disappeared.

At the one‑month postpartum follow-up, the mother demonstrated good recovery with complete resolution of hydronephrosis, supporting the diagnosis of transient ureteral compression from the gravid uterus at 35 weeks. Follow‑up chest MRI confirmed persistent occlusion of the embolized PAVM (Figure 7). The patient is being followed up at the otolaryngology department for epistaxis, but no difficulties in stopping the bleeding have been noted.

Following this delivery, the patient is now the mother of three sons and two daughters. Once a pathogenic variant associated with HHT is identified within a family, genetic testing can facilitate early diagnosis among at-risk relatives. In the present case, the patient was provided with counseling regarding genetic testing following notification of her diagnosis, but she has not requested genetic testing for herself or her children.

## Discussion

Maternal risks associated with unrecognized HHT

This case highlights the profound maternal risk associated with unrecognized hereditary hemorrhagic telangiectasia in pregnancy, demonstrating that catastrophic rupture of a large pulmonary arteriovenous malformation can occur in the absence of appropriate pre-pregnancy evaluation, and that rapid multidisciplinary endovascular intervention can be lifesaving, even in the setting of a dichorionic diamniotic twin gestation.

HHT is a multisystem disease with three main features: recurrent epistaxis, multiple telangiectasias, and visceral arteriovenous malformations [2]. Telangiectasias typically appear on the lips, tongue, buccal and gastrointestinal mucosa, face, fingers, etc., and frequently develop in the lungs, liver, and brain as major arteriovenous malformations [1]. The mean age of onset is 12 years, and almost all patients present with the condition by the age of 40 years [1, 3]. PAVMs are right-to-left shunts and can cause hypoxemia in some patients; however, few patients complain of respiratory symptoms [3, 4]. PAVMs must be closed to prevent strokes, transient ischemic attacks, cerebral abscesses, and other complications [1]. In the present case, the patient was diagnosed with HHT at 32 years of age after rupture of a PAVM during pregnancy, highlighting a missed opportunity for earlier recognition. Overall, PAVMs affect approximately 50% of patients with HHT. Many of the complications reported during pregnancy are associated with PAVMs, underscoring their importance in obstetric management.

Although spontaneous rupture during pregnancy is rare, the hemodynamic and hormonal changes of gestation, including progesterone-mediated venodilation and increased circulating blood volume, can promote enlargement or rupture of PAVMs. Despite having undergone three abdominal cesarean sections in Afghanistan, the patient had never undergone chest radiography. Although HHT had been suspected during her previous pregnancies because of recurrent epistaxis and mucocutaneous telangiectasia, the diagnosis was not confirmed. Twin pregnancies impose even greater hemodynamic stress than singleton pregnancies, potentially compounding the risk of PAVM rupture in this patient.

Serious complications during pregnancy include hemothorax and hematoma due to PAVMs, severe hypoxemia, worsening of the right-to-left shunt of the PAVMs, intracranial hemorrhage due to rupture of a cerebral arteriovenous malformation, acute liver failure due to hepatic arteriovenous malformation, heart failure, and biliary necrosis [4-6]. As previously noted, severe complications can arise during pregnancies with HHT; therefore, early treatment before pregnancy should be considered for women of childbearing potential [1,3]. If possible, patients should be screened before pregnancy, and those diagnosed with PAVMs are recommended to undergo embolization of any malformations [1].

Rationale for emergency embolization during pregnancy

Prior studies consistently demonstrate that pregnant patients with unrecognized HHT are at increased risk for severe complications, particularly when PAVMs remain untreated. Pregnant patients with PAVMs are reported to have a mortality rate of 1% and must be treated once they have been informed that their pregnancy is high risk [4]. Dupuis et al. reviewed 1577 pregnancies in 630 patients with HHT and reported that 2.7% to 6.8% of patients developed serious HHT complications, predominantly during the second and third trimesters in those who had not been diagnosed or screened [6]. These findings are consistent with the present case, in which the absence of prior evaluation allowed a PAVM to progress until catastrophic rupture in mid-pregnancy. Collectively, this data underscores that unrecognized HHT confers substantial maternal risk, particularly when visceral AVMs remain undetected at the onset of pregnancy. When PAVMs are identified during pregnancy, embolization during or after the second trimester is recommended to minimize fetal radiation exposure [1]. Deterministic effects of fetal radiation exposure occur only at relatively high doses, and the associated risks decrease with advancing gestational age. From 16 weeks of gestation to term, a decline in intelligence quotient has been reported at exposures around 100 mGy, whereas growth retardation is observed at doses exceeding 250 mGy [7]. These thresholds indicate that, in most clinical scenarios, diagnostic imaging performed with appropriate dose management is unlikely to pose significant fetal risk, even in emergency settings.

The standard treatment is pulmonary embolization by catheterization. Embolization is technically difficult in PAVMs with feeding artery diameters of less than 2 to 3 mm [4]. Many patients with HHT have multiple PAVMs, only some of which are considered suitable for embolization. In such cases, the treatment objective is not complete closure of the PAVMs, but rather improvement of the shunt [4]. Gershon et al. reviewed 26 cases in which serious complications arose due to PAVMs that developed during pregnancy, including four cases in which transcatheter embolization was performed during pregnancy [8]. Very few studies have reported transcatheter embolization being performed during pregnancy; the reports, including the present case, are listed in Table 2 [9-13].

**Table 2:** Reported cases of transcatheter embolotherapy for maternal pulmonary arteriovenous malformation during pregnancy * information not available PAVM - pulmonary arteriovenous malformation; HHT - hereditary hemorrhagic telangiectasia

Author, year	Age	Gravida/parity	Gestational age	Presenting symptoms	PAVM complications	HHT	Maternal outcome	Fetal outcome
Waring et al., 1990 [9]	27	G3P2	26 w	Hemothorax	Hemothorax	+	Alive	Alive
Bevelaqua et al., 1992 [10]	24	—	24 w	Hemothorax	Hemothorax	+	Alive	Alive
Esplin et al., 1997 [11]	36	G1P1	24 w	Intrapulmonary hemorrhage	Intrapulmonary hemorrhage	Not applicable	Alive	Alive (postpartum PAVM resection required)
Desai et al., 1997 [12]	26	G3P2	20 w	Hemoptysis	Hemoptysis	+	N/A*	N/A*
Kamimura et al., 2021 [13]	27	G4P1	30 w	Hemothorax	Hemothorax	+	Alive	Alive
Present case	32	G4P3	20 w	Hemothorax	Hemothorax	+	Alive	Alive

In some cases, performing transcatheter embolization is difficult because of the patient’s general condition [6, 12]. Surgery should be considered for patients at high risk of complications, for example, those with a history of recurrent ischemic attacks [4]. PAVM rupture can be accompanied by symptoms such as lung parenchymal, bronchial, or intrathoracic bleeding, hemothorax, pulmonary hemorrhage, and hemoptysis. Li et al., who collected 31 cases of major PAVMs accompanied by hemothorax, reported aneurysmal sac diameters exceeding 30 mm in surgically treated cases [14]. They also reported that, in four of 18 patients who were treated with transcatheter embolization, the embolization resulted in no improvement, and surgical treatment was required [14]. Although the aneurysmal sac size in our patient was relatively large at 30 mm, only a single sac was present. Current treatment criteria generally recommend embolization for PAVMs with a feeding artery diameter of ≥2-3 mm, as these lesions carry a meaningful risk of complications. In our patient, the aneurysmal sac measured 30 mm, clearly exceeding conventional treatment thresholds and indicating a substantially elevated risk of rupture. The size and morphology of the lesion, therefore, strongly supported the decision for urgent transcatheter embolization, which was both clinically appropriate and consistent with established management guidelines. Given these considerations, the clinical decision-making process required careful evaluation of both maternal and fetal risks. Therefore, we deemed transcatheter embolization the appropriate treatment. In this case, urgent embolization at 20 weeks of gestation was selected based on several critical clinical factors. The aneurysmal sac measured 30 mm, far exceeding treatment thresholds and indicating a high risk of re‑rupture. The patient also showed hemodynamic instability due to severe anemia and hemothorax, necessitating prompt intervention. A multidisciplinary team - including obstetrics, interventional radiology, respiratory medicine, and pediatrics - concluded that immediate embolization offered the safest approach. This case highlights that timely endovascular treatment can be justified even in mid‑pregnancy when maternal risk is substantial and fetal radiation exposure can be minimized with appropriate precautions. A surgical approach was considered in the event of repeated rupture following embolization or if no improvement in symptoms was observed. Although embolization for PAVM rupture during pregnancy has been described, previously reported cases rarely involved dichorionic diamniotic twin gestations or presentation with profound hemorrhagic shock in the mid-second trimester. The large diameter of the PAVM (30 mm) and the use of compression hemostasis to treat hemorrhagic pleural effusion after rupture are also thought to have contributed to the success of the embolization. Chronic maternal hypoxemia, hypovolemia, and maternal death can lead to intrauterine growth restriction or intrauterine fetal death [5]. However, in the present case, good outcomes were achieved with transcatheter embolization and transfusion.

Post‑treatment surveillance and genetic implications

As PAVMs can reopen or enlarge even after transcatheter embolization, the mother should undergo regular follow-up examinations with contrast echocardiography and chest CT [2, 4]. With regard to post-treatment surveillance, current international guidelines recommend follow-up imaging 6-12 months after embolization to assess for recanalization or the development of new PAVMs, followed by surveillance every three to five years in adults. Thin-slice contrast-enhanced chest CT is considered the modality of choice, whereas contrast echocardiography may be used when radiation exposure is a concern. Incorporating these surveillance strategies is essential for preventing delayed complications, particularly in patients with HHT who remain at lifelong risk of developing new or recurrent PAVMs [2]. Regarding long-term outcomes after embolization, Fish et al. reported that HHT patients with PAVMs who were followed for an average of 16.3 years rarely experienced re-rupture, demonstrating the durable efficacy of embolization [15]. This evidence underscores the importance of careful postprocedural follow-up in the present case as well.

Although our patient did not request genetic testing and none was performed, the autosomal dominant inheritance pattern of HHT underscores the need for appropriate evaluation of both the patient and her children. Three causative genes - ENG, ACVRL1, and SMAD4 - have been identified [1]. Genetic testing plays a central role in confirming a clinical diagnosis, establishing a diagnosis in symptomatic individuals, and identifying affected family members when a pathogenic variant is known. For symptomatic individuals, diagnostic testing can clarify future risks and guide management strategies. For asymptomatic relatives, early identification of a pathogenic variant enables tailored surveillance and may help avoid medications or exposures that could precipitate symptoms. Given the hereditary nature of HHT, proactive family screening is strongly recommended to ensure early detection of visceral AVMs and to prevent potentially life-threatening complications. In the context of this case, timely genetic evaluation might have facilitated earlier recognition of HHT and potentially prevented the catastrophic presentation during pregnancy.

In summary, this case highlights the substantial maternal risk posed by unrecognized HHT in pregnancy, demonstrating that catastrophic PAVM rupture can occur without appropriate pre-pregnancy evaluation. The favorable outcome in our patient was achieved through rapid multidisciplinary assessment and timely endovascular intervention. This experience reinforces the importance of early, coordinated perinatal management to optimize maternal outcomes in cases of AVM rupture during pregnancy.

## Conclusions

In conclusion, this case illustrates the substantial maternal risk associated with unrecognized hereditary hemorrhagic telangiectasia (HHT) during pregnancy, demonstrating that catastrophic pulmonary arteriovenous malformation (PAVM) rupture can occur in the absence of appropriate pre‑pregnancy evaluation. The convergence of undiagnosed HHT, rupture of a large PAVM at 20 weeks of gestation, and the requirement for urgent endovascular intervention in a dichorionic diamniotic twin pregnancy underscores the exceptional rarity of this presentation. The favorable maternal and neonatal outcomes highlight the lifesaving importance of rapid multidisciplinary coordination and timely embolization. This case further underscores the need to maintain a high index of clinical suspicion for HHT, particularly in women of reproductive age, to facilitate timely diagnosis and avert life‑threatening complications. Moreover, routine screening for HHT in individuals with suggestive clinical features and pre‑pregnancy evaluation for women with possible HHT, such as those with recurrent epistaxis, may enable the identification of PAVMs and other vascular lesions before conception, thereby reducing the risk of catastrophic complications.
